# Green Alga *Ulva* spp. Hydrolysates and Their Peptide Fractions Regulate Cytokine Production in Splenic Macrophages and Lymphocytes Involving the TLR4-NFκB/MAPK Pathways

**DOI:** 10.3390/md16070235

**Published:** 2018-07-11

**Authors:** Raúl E. Cian, Cristina Hernández-Chirlaque, Reyes Gámez-Belmonte, Silvina R. Drago, Fermín Sánchez de Medina, Olga Martínez-Augustin

**Affiliations:** 1 Instituto de Tecnología de Alimentos, CONICET, FIQ-UNL, 1 de Mayo 3250, 3000 Santa Fe, Argentina; rec_704@yahoo.com.ar (R.E.C.); dragosilvina@hotmail.com (S.R.D.); 2 Department of Biochemistry and Molecular Biology II, CIBERehd, School of Pharmacy, Instituto de Investigación Biosanitaria ibs.GRANADA, University of Granada, 18071 Granada, Spain; cristinahech@hotmail.com (C.H.-C.); omartine@ugr.es (O.M.-A.); 3 Department of Pharmacology, CIBERehd, School of Pharmacy, Instituto de Investigación Biosanitaria ibs.GRANADA, University of Granada, 18071 Granada, Spain; reyesgb@ugr.es

**Keywords:** bioactive peptides, green seaweeds, interleukin 10, TLR2, TLR4, NFκB

## Abstract

Hydrolysates of food protein sources have immunomodulatory effects, which are of interest for use as functional foods. In this study, we have characterized the immune regulatory effect on rat splenocytes, macrophages and T lymphocytes of *Ulva* spp. hydrolysates and their peptide fractions with or without in vitro gastrointestinal digestion and/or ultrafiltration. IL-10 was induced in almost all conditions and cell types obtained from wild type animals. The induction was in general increased by ultrafiltration and in vitro gastrointestinal digestion. TNF was also induced in basal conditions. In turn, TNF and IFN-γ production was attenuated by the hydrolysate products in lipopolysaccharide or concanavalin A immune stimulated cells. Inhibitors for the activation of NFκB, MAPK p38 and JNK inhibited IL-10 induction in rat splenocytes. The response was dramatically attenuated in TLR4^−/−^ cells, and only modestly in TLR2^−/−^ cells. Food peptides from *Ulva* spp. genus exert anti-inflammatory effects in immune cells mediated by TLR4 and NFκB. Similarity with the immunomodulatory profile of protein hydrolysates from other sources suggests a common mechanism.

## 1. Introduction

Genus *Ulva* is a group of edible green algae that are widely distributed along the coasts worldwide [1]. *Ulva* spp. have an interesting chemical composition that makes their commercial exploitation attractive to produce functional or health-promoting foods [2]. Particularly, *Ulva* spp. have high levels of protein (15–33% *w w*^−1^), thus constituting a potential source of bioactive peptides [3]. In this regard, many algae-derived bioactive peptides have been obtained by in vitro enzymatic hydrolysis [4], and have been shown to exert many physiological functions, including antioxidant, anticancer, antihypertensive, anti-atherosclerotic, and immunomodulatory effects [5]. Thus, Ko et al. (2012) reported that the oral administration of Val-Glu-Gly-Tyr peptide, purified from green alga *Chlorella ellipsoidea* hydrolysates, significantly decreased systolic blood pressure in spontaneously hypertensive rats. Morris et al. found that the oral administration of a protein hydrolysate from green alga *Chlorella vulgaris* activates both innate and specific immune responses, including a marked increase in the lymphocyte number, the production of T cell-dependent antibody responses, and the reconstitution of delayed-type hypersensitivity responses in undernourished Balb/c mice [6]. Similarly, a mitogenic hexapeptide (EDRLKP) has been isolated from *Ulva* spp. [7] and shown to be involved in modulation of cell proliferation-associated molecules, namely proteoglycans and glycosaminoglycan, in human foreskin fibroblasts.

In vitro studies searching for cellular mechanisms of action of red algae-derived immunomodulatory hydrolysates have frequently used splenocytes, macrophages and T lymphocytes. In these studies, a common feature is an anti-inflammatory effect of bioactive peptides mediated by the induction of IL-10 production, both in basal and stimulated conditions. Other frequently reported actions include the inhibition of TNF and IFN-γ production in lipopolysaccharide (LPS) or concanavalin A (ConA) stimulated cells [8,9,10]. The molecular mechanism of action of red algae-derived immunomodulatory peptides involved in these effects features the modulation of NFκB and several MAPKs. However, the molecular mechanism of action of green algae-derived peptides (signal transduction pathways or receptors involved) has not been studied.

Toll-like receptors (TLRs) are innate immunity receptors. The TLR ligands initially described were bacterial and viral components/antigens, but a wide range of other molecules can also activate them. For example the TLR4 receptor, initially described to bind LPS, is currently known to be also activated by endogenous molecules such as fibrinogen, heparan sulfate, and by food-derived molecules such as cabbage methylsulfinylpropyl isothiocyanate (iberin) or onion quercetin and quercetin 4′-*O*-β-glucoside [11,12].

Here we have studied the immunoregulatory effect of *Ulva* spp. hydrolysates and their peptide fractions on murine splenocytes, macrophages and T lymphocytes. In addition, we have used TLR2 and TLR4 knockout mice to ascertain the involvement of these receptors, and finally we have assessed the implication of the NFκB and MAPKs pathways. Our results show that peptides and hydrolysates obtained from *Ulva* spp. have in vitro immunomodulatory actions consistent with an anti-inflammatory effect, which is related to ligation of TLR2/4 and involves NFκB and the MAPKs p38 and JNK. In addition, this study contributes to the accumulating evidence supporting the hypothesis of a common, non-specific effect of food peptides in the regulation of the immune response.

## 2. Results

### 2.1. Characterization of Hydrolysates and Their Peptide Fractions

In the present study, *Ulva* spp. green seaweed was sequentially hydrolyzed with Purazyme (P) + Flavourzyme (F) or Alkaline protease-Protex 6L (A) + Flavourzyme (F). The hydrolysates obtained were named PF and AF, respectively. The extent of protein degradation by proteolytic enzymes was estimated by assessing the degree of hydrolysis (DH), which was higher for AF than for PF (23.6 ± 2.2% vs. 16.7 ± 1.7%, respectively) indicating that the AF system was more efficient on *Ulva* spp. proteins. Consistent with this, the PF hydrolysate showed a high fraction of peptides with molecular weight (MW) of 3150 kDa, while the main fraction in AF was 2160 kDa (Supplementary Figure S1A,B, respectively). In both hydrolysates a peak of ≈300 Da was observed, indicating the presence of low MW peptides generated by the F enzyme.

As expected, ultrafiltration of PF and AF hydrolysates resulted in the selection of peptides with MW <1 kDa (Supplementary Figure S1C,D, respectively). Both ultrafiltered fractions (named PFU and AFU) showed peptides of ≈1100 and ≈600 Da. However, the proportion of low MW peptides (600 Da) and free amino acids (150 Da) of AFU was higher than that obtained with PFU (*P* < 0.05), which agrees with the DH found for each hydrolysate.

The PF hydrolysate subjected to in vitro gastrointestinal digestion and ultrafiltration (PFDU) showed a similar FPLC gel filtration profile to that of PFU (Supplementary Figure S1E), indicating that peptides from PF were resistant to in vitro pepsin and pancreatin hydrolysis. However, the AFDU profile showed a higher reduction of the area corresponding to peptide fractions (1100 kDa) than PFU, while the proportion of low MW peptides (600 Da) and free amino acids (150 Da) was increased (Supplementary Figure S1F). Thus, in vitro gastrointestinal digestion affected the MW distribution of peptides from the AF hydrolysate, indicating the generation of new species from the 1100 Da peptides.

### 2.2. Green Alga Ulva spp. Hydrolysate and Their Peptide Fractions Induce the Expression of the Anti-Inflammatory Cytokine IL-10 in Spleen Cells

**Figure 1:**
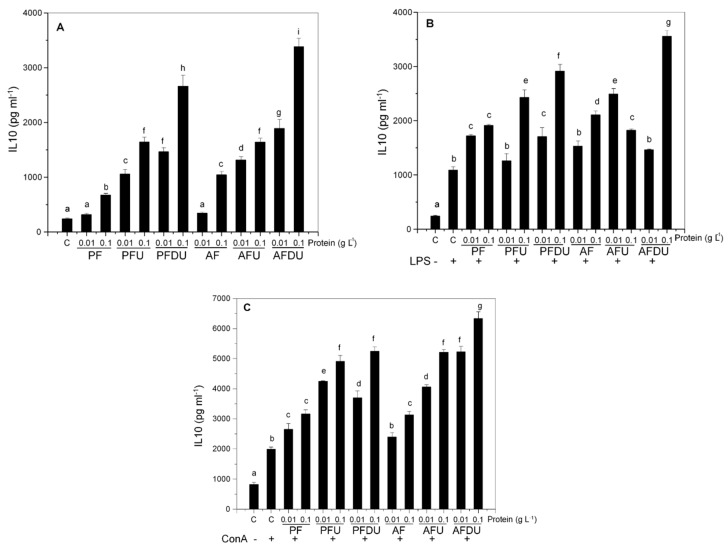
Effect of *Ulva* spp. hydrolysate products on the production of IL-10 by rat splenocytes. Splenocytes were cultured with *Ulva* spp. products (0.01 and 0.1 g L^−1^ of protein) in the absence (**A**), or presence of bacterial LPS (1 µg mL^−1^) (**B**), or the presence of ConA (5 µg mL^−1^) (**C**). Data are expressed as mean ± SD. Means for a variable without a common letter are significantly different (*P* < 0.05).

The addition of all the products to rat spleen cells increased the production of IL-10 in basal conditions (Figure 1A). The ultrafiltration and in vitro gastrointestinal digestion followed by ultrafiltration potentiated this effect. This may be the result of specific peptide concentration and the release of new peptides. The hydrolysates obtained with Alkaline protease-Protex 6L + Flavourzyme (AF hydrolysates) had a slightly higher impact on this parameter than those made with Purazyme + Flavourzyme (PF hydrolysates), in agreement with the higher DH of the former. As expected, the stimulation with LPS or ConA induced the production of IL-10. The addition of hydrolysates to LPS or ConA stimulated cells further increased the concentration of IL-10 in the culture media (Figure 1B,C, respectively). As observed in basal conditions, ultrafiltration and in vitro gastrointestinal digestion followed by ultrafiltration potentiated the effect.

### 2.3. Green Alga Ulva spp. Hydrolysates and Their Peptide Fractions Regulate the Expression of Proinflammatory Cytokines TNF and IFN-γ in Spleen Cells

**Figure 2:**
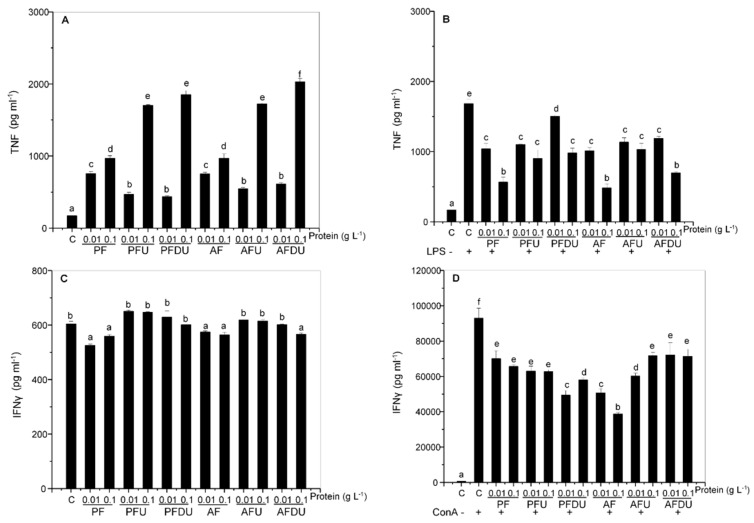
Effect of *Ulva* spp. hydrolysate products on the production of TNF and IFN-γ by rat splenocytes. Splenocytes were cultured with *Ulva* spp. products (0.01 and 0.1 g L^−1^ of protein) in the absence (**A**,**C**), or presence of bacterial LPS (1 µg mL^−1^) (**B**), or of ConA (5 µg mL^−1^) (**D**). Data are expressed as mean ± SD. Means for a variable without a common letter are significantly different (*P* < 0.05).

*Ulva* ssp. hydrolysates and their peptide fractions induced the production of TNF in basal conditions in rat splenocytes (Figure 2A). In this case, only ultrafiltration potentiated the effect, while in vitro gastrointestinal digestion followed by ultrafiltration had little or no additional effect. No differences were observed between PF or AF hydrolysates.

When cells were stimulated with LPS, TNF production was induced but, interestingly, the addition of all products inhibited rather than enhanced its expression, an effect that was concentration dependent in most cases, and was not further augmented by ultrafiltration or in vitro gastrointestinal digestion followed by ultrafiltration (Figure 2B). Thus, inhibition of TNF expression was highest with 0.1 g L^−1^ of hydrolysates (PF and AF) and with 0.1 g L^−1^ of the AFDU fraction.

In contrast to TNF and IL-10, the production of IFN-γ in basal conditions was either not affected or minimally inhibited by all products (Figure 2C). The magnitude of this effect, although statistically significant, was small. When ConA was added to the culture medium, all the products inhibited the production of IFN-γ, AF and PDFU being most effective (58–38%, Figure 2D).

### 2.4. Macrophages and T Lymphocytes Contribute to the Anti-Inflammatory Effect of Green Alga Ulva spp. Hydrolysates and Their Peptide Fractions

**Figure 3:**
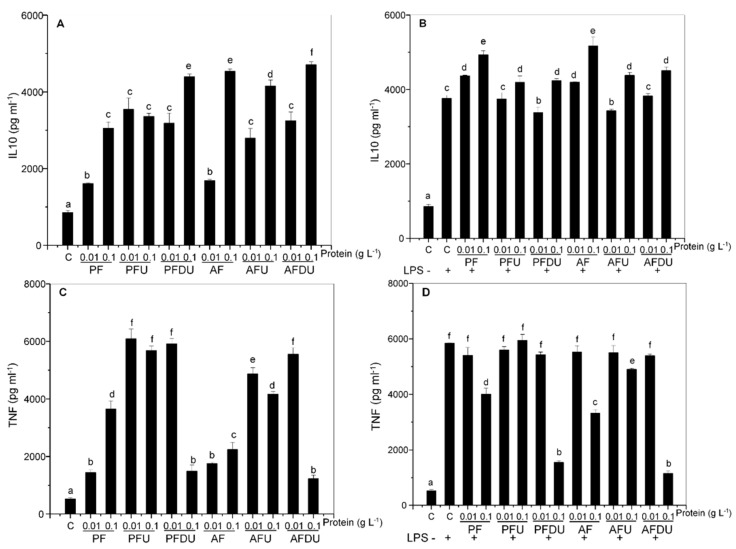
Effect of *Ulva* spp. hydrolysate products on the production of IL-10 and TNF by rat macrophages. Macrophages were cultured with *Ulva* spp. products (0.01 and 0.1 g L^−1^ of protein) in the absence (**A**,**C**), or presence of bacterial LPS (1 µg mL^−1^) (**B**,**D**). Data are expressed as mean ± SD. Means for a variable without a common letter are significantly different (*P* < 0.05).

**Figure 4:**
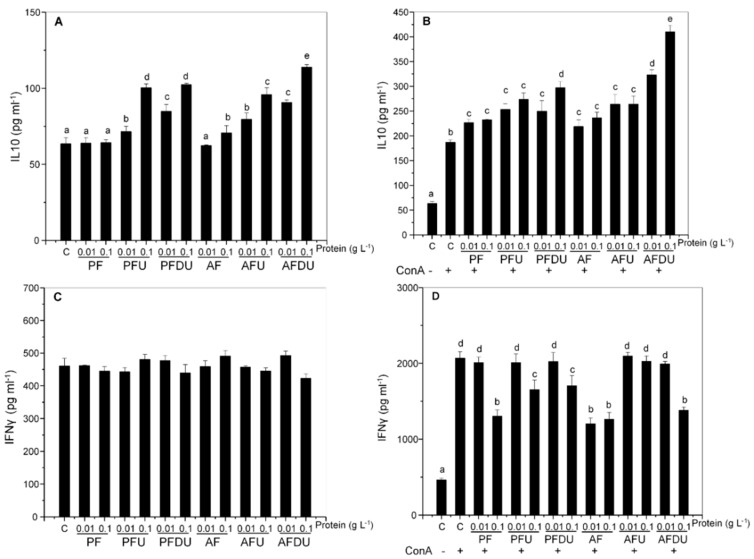
Effect of *Ulva* spp. hydrolysate products on the production of IL-10 and IFN-γ by rat lymphocytes. Lymphocytes were cultured with *Ulva* spp. products (0.01 and 0.1 g L^−1^ of protein) in the absence (**A**,**C**), or presence of ConA (5 µg mL^−1^) (**B**,**D**). Data are expressed as mean ± SD. Means for a variable without a common letter are significantly different (*P* < 0.05).

Macrophages and lymphocytes are both present in splenic primary cultures. Therefore, we aimed to characterize the observed effects in these cell types isolated from rat spleen (Figure 3 and Figure 4). Effects of the *Ulva* ssp. hydrolysates and their peptide fractions on macrophages cultured in basal conditions were similar to those observed in splenocytes, with induction of IL-10 production by all of them. The effect was maximal with PFDU and AFDU, but it was already substantial with the PF and AF hydrolysates, and was again in most cases concentration dependent (Figure 3A). Ultrafiltration augmented the IL-10 inducing action of the lower but not the higher concentration of PF/AF, i.e., a sensitizing effect. In contrast, in vitro gastrointestinal digestion followed by ultrafiltration had a smaller impact and appeared to push up the effect of the higher concentration of the hydrolysate, at least in the case of PFDU vs. PFU. Stimulation with LPS increased the production of IL-10, which was further increased by the addition of all the products, but to a relatively lower degree and not in all the conditions tested (Figure 3B). Globally, when compared, the magnitude of the effects on IL-10 was higher in spleen cells than in macrophages, perhaps because the latter produce higher amounts of IL-10 in both basal and LPS stimulated conditions.

TNF production was, as in splenocytes, induced in rat macrophages in basal conditions by the addition of all the products. Also, ultrafiltration and in vitro gastrointestinal digestion followed by ultrafiltration enhanced again these effects, except for PFDU/AFDU at 0.1 g L^−1^ (Figure 3C). In this cell type the effect of the AF products was somewhat lower than that of PF products. As in splenocytes, LPS evoked TNF production was generally inhibited by the hydrolysates, with the notable exception of PFU/AFU, which had little or no effect (Figure 3D). The inhibitory effect was globally lower than with splenocytes, and was maximal with PFDU/AFDU, followed by PF/AF, at 0.1 g L^−1^ in all cases.

Culturing rat T lymphocytes with the hydrolysate products resulted in a modest up-regulation of basal IL-10 release by double digested and ultrafiltered products, with little or no effect of PF/AF (Figure 4A). Similar enhancing effects were observed after ConA stimulation, but in this case all the products were active (Figure 4B).

In contrast, no effect was observed on basal IFN-γ secretion (Figure 4C) and, as in splenocytes, an inhibition was generally observed in ConA stimulated cells cultured with the products (Figure 4D). This inhibition was significant for all the products at the higher concentration assayed (and AF at both concentrations), except for AFU, which had no effect.

### 2.5. Effect of Green Alga Ulva spp. Hydrolysate and Their Peptide Fractions by Splenocytes Are Mediated through TLR4 and TLR2

**Figure 5:**
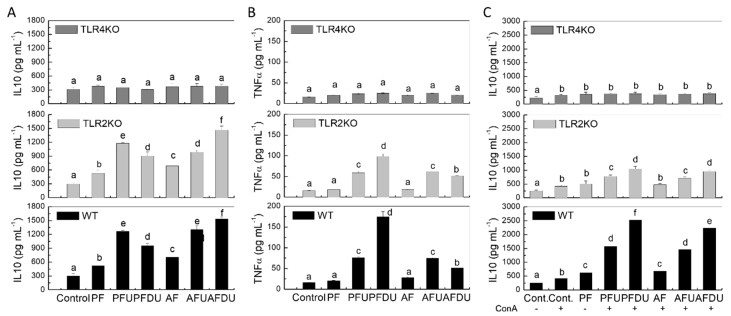
Effect of *Ulva* spp. hydrolysate products on IL-10 and TNF production by splenocytes from WT, TLR2 KO and TLR4 KO mice. Splenocytes were cultured with *Ulva* spp. products (0.01 and 0.1 g L^−1^ of protein) in the absence (**A**,**B**), or presence of ConA (5 µg mL^−1^) (**C**). Data are expressed as mean ± SD. Means for a variable without a common letter are significantly different (*P* < 0.05).

Since TLR2 and TLR4 are promiscuous receptors, in that they have significant affinity for a variety of different ligands, including a wide range of food-derived products, we obtained splenocytes from TLR2 and TLR4 knockout mice and cultured them with the products in basal conditions and in the presence of ConA (Figure 5). While the addition of the products to splenocytes from wild type mice, as observed for rat splenocytes above, induced the production of IL-10 and TNF in basal conditions, no effect was observed when splenocytes from TLR4 KO mice were used, indicating that stimulation of TLR4 is needed for the stimulated production of both cytokines. In TLR2 KO mice the production of IL-10 showed no changes whatsoever compared to WT mice, while TNF was inhibited, but without altering the overall profile compared with the controls. Therefore, these results indicate that TLR2 is not involved in the IL-10 response and that it has only a minor role in TNF production (Figure 5B).

We also assessed the involvement of TLR2/4 in the enhancement of IL-10 release by the hydrolysates under ConA stimulation. As in the case of the rat, mouse splenocytes showed an increased IL-10 production in these conditions (Figure 5C). This profile was largely preserved in cells obtained from TLR2 KO mice, while all tested protein/hydrolysate products failed to modulate IL-10 output in TLR4 KO cells, indicating that TLR4 is also required in ConA stimulatory conditions.

### 2.6. NFκB Activation Is Needed for IL-10 Induction by Green Alga Ulva spp. Hydrolysate and Peptide Fractions, with Secondary Involvement of JNK and p38 MAPK

**Figure 6:**
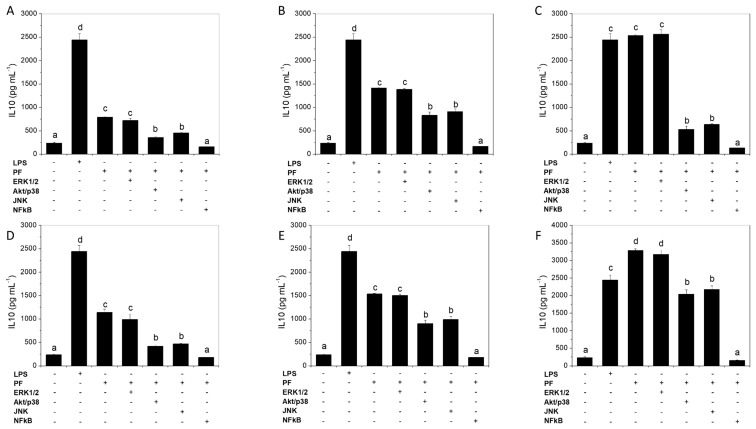
Effect of MAP kinase and NFκB inhibitors on IL-10 by rat splenocytes stimulated with *Ulva* spp. hydrolysate products. Cells were pre-incubated for 1 h with the signal transduction inhibitors. LPS (1 µg mL^−1^) was used as reference. The products tested are PF (**A**), PFU (**B**), PFDU (**C**), AF (**D**), AFU (**E**), AFDU (**F**). Data are expressed as mean ± SD. Means for a variable without a common letter are significantly different (*P* < 0.05).

NFκB activation induces cytokine expression. Signal transduction pathways that lead to augmented cytokine expression and to NFκB activation include the MAPKs JNK, ERK and p38. We used inhibitors of NFκB and MAPKs activation to explore signal transduction pathways downstream of TLR4 (Figure 6). The inhibition of NFκB with Bay 11-7082 abrogated the IL-10 induction evoked by the products. Inhibition of p38 MAPK and JNK partially inhibited the response, while ERK seemed not to be involved. It is interesting to note that the response to the inhibitors was homogeneous among groups, suggesting common signal transduction pathways independently of the product used to stimulate IL-10 production.

## 3. Discussion

Inflammatory diseases represent a very significant group of human morbidities, affecting a large part of the population. Clinical management is often difficult, particularly in those instances where the inflammatory condition is chronic, and the causative element has not been identified or cannot be eliminated: rheumatoid arthritis, psoriasis, uveitis, inflammatory bowel disease, etc. While undoubtedly the core of therapy is pharmacological, there is a strong and increasing interest in pursuing also a nutritional angle in the form of nutraceutics or functional foods. The latter may be defined as food similar to conventional food in appearance, intended to be consumed as part of a normal diet, and containing biologically active compounds which offer potential for enhanced health or reduced risk of disease [13]. Nutraceuticals may be viewed as the ‘active’ components of functional foods, but a consensus definition has not been reached [14]. The goal is to coadyuvate to the overall management of inflammatory diseases, playing a supportive role in the context of medical therapy.

Protein hydrolysates and peptides have immunomodulatory properties as shown by several different studies, including our own [11,15,16,17,18,19]. These effects depend largely on their ability to remain intact after digestive process and reach the target organ. The peptides that resist the digestive process and arrive intact in the intestine can have a local function or may be able to cross the epithelium, enter the bloodstream, and have a systemic effect [20,21]. Our aim was to study the immunomodulatory activity of peptides obtained from *Ulva* spp. with and without in vitro gastrointestinal digestion, since these widely distributed algae used for human consumption are a potential source of immunoregulatory peptides, whose activity could be modified by the action of digestive enzymes. It should be noted that we cannot rule out the contribution of nonpeptide molecules to the observed effects; glycans in particular as lipids are removed in the extraction process. However, in a control experiment it was shown that precipitation with 100% trichloroacetic acid resulted in complete absence of bioactivity (Supplementary Figure S3). Thus, the effects reported in our study are accounted for mainly by peptides and proteins present in the different products, including glycoproteins and/or any protein-bound molecules that may co-precipitate in an acidic environment.

Our results confirm the immunomodulatory activity of these products, generated under different conditions. Specifically, peptides from *Ulva* spp. induce the production of the anti-inflammatory cytokine IL-10 in both basal and stimulated conditions in splenocytes and in splenic macrophages and lymphocytes. This effect was most pronounced in splenocytes, followed by macrophages and finally T cells. This may be simply the consequence of the different IL-10 production rates in the respective cell populations (i.e., mixed spleen cells secrete higher amounts than isolated macrophages or T cells).

In contrast, while TNF release is enhanced in quiescent splenocytes and macrophages, this proinflammatory cytokine is downregulated in LPS treated cells. On the other hand, IFN-γ secretion is unchanged in quiescent splenocytes and T cells, and it is downregulated under ConA stimulatory conditions. Therefore, the overall profile is consistent with an anti-inflammatory activity, although TNF upregulation might conceivably lead to inflammatory responses as well. In this regard it is worth noting that other nutraceutical products with potentially immunostimulatory effects such as non-absorbable glucids or bovine glycomacropeptide are nontoxic when given orally and in fact have intestinal anti-inflammatory effects, which is attributed at least in part to reinforcement of intestinal barrier function [22,23,24,25,26,27]. In vivo experiments will be required to answer this question definitively.

Of note, this array of immunomodulatory activity was reproduced in mouse cells, which allowed us to examine the involvement of TLR2 and TLR4 by using primary cell cultures from the respective KO mice. Although we cannot ascertain from our experiments if peptides or proteins from algae are direct TLR4 ligands, the lack of IL-10 and TNF induction in cells derived from TLR4 but not TLR2 KO mice points to the specific involvement of TLR4 in the mechanism of action. In addition, attenuation of the TNF response in macrophages in TLR2 KO mice suggests that this receptor is also involved to a certain extent. Consistent with TLR2/4 signal transduction through the NFκB pathway, IL-10 induction is completely abrogated by Bay 11-7082, and by inhibitors targeting the p38 and JNK MAPKs, indicating that the signaling pathway depends at least partially on p38 and JNK activation.

Effects of hydrolysates and peptide fractions from different food sources on IL-10, TNF and IFN-γ production in splenocytes, macrophages and T lymphocytes have been previously described and reviewed [28]. Notably, in these studies the main findings are, broadly, cytokine induction in intact macrophages, with inhibition under stimulatory conditions. In general, results are difficult to compare due to differences in experimental conditions and the algae preparations tested. Nevertheless, our research group has previously described quite similar results, testing protein hydrolysates from *Phorphyra columbina* sequentially digested with fungal proteases and Flavourzyme [8,9,10,28], under equivalent experimental conditions. Thus, induction of IL-10 in the 3 cell types used in the present study, together with inhibition of proinflammatory cytokines (TNF when stimulated with LPS and IFN-γ under ConA stimulation) were described. Protein hydrolysates from other sources such as cow’s milk have also been shown to induce IL-10 and TNF in circulating monocytes [16]. Although in this article the authors found intact cow’s milk proteins to have no effect, this has not been the case with intact proteins from *Phorphyra columbina* or *P. yezoensis*, which reportedly are immunoactive [8,9,10,28,29]. Thus, increased IL-10 production is observed when a protein concentrate of *Phorphyra columbina* is incubated with macrophages or T lymphocytes, while inhibited proinflammatory TNF/IL-1β secretion has been reported for PGP (*P. yenzoensis* GlycoProtein), in LPS stimulated macrophages.

Further, the mechanism of action appears to be similar as well. For instance, *P. yezoensis* PGP protein (along with various glucidic compounds obtained from this alga) exerts immunomodulatory effects through TLR4 [29]. The effects of protein hydrolysates from *Phorphyra columbina* alluded to before [8,9,10,28,29] are mediated by the TLR4/NFκB pathway. Such a convergent mechanism of different peptides/hydrolysates is consistent with the properties of TLRs in general, and TLR4 in particular, as they are rather promiscuous, in that they may be engaged by a wide variety of ligands. The current list of ligands for TLR4 includes calprotectin, heat-shock proteins, saturated fatty acids, non-digestible oligosaccharides, quercetin, bovine glycomacropeptide-derived peptides, and so forth [11,12,25,30]. Considered globally, the evidence available from this and other studies suggests that protein hydrolysates and peptides may have broadly similar immunomodulatory activities which appear to share a common basic mechanism of action. This does not exclude the possibility of differences of course. In the context of the present study, the processing of the primary hydrolysates by either ultrafiltration or double digestion potentiated the effect of the products on IL-10 and TNF production in quiescent cells, indicating that changes in the protein fragments present in the mix do affect the final result. This may be related to an increased peptide concentration, the size of the peptides or the release of new peptides. Another possibility is the release of protein-associated glycans. However, it is noteworthy that this had a relatively minor influence on the result.

Our results support the possible application of *Ulva* spp. hydrolysates as immunomodulatory nutraceuticals. The bioactive components have been proven to retain activity at acidic pH and therefore they can be presumed to be essentially unaffected by gastric digestion. However, as mentioned above, it is essential to carry out additional studies in the future to establish their utility in vivo, which depends on their capacity to cross the epithelial barrier and access the subepithelial milieu or more distal sites.

In conclusion, hydrolysates obtained from *Ulva* spp. exert immunomodulatory actions in vitro which are consistent with an anti-inflammatory effect, and which depend on TLR4 and the NFκB/p38/JNK pathway. This profile is reminiscent of the biological activities shown by other protein hydrolysates from different sources, suggesting a possible common basic effect and mechanism.

## 4. Materials and Methods

### 4.1. Reagents

Alkaline protease-Protex 6L (A) and Purazyme^®^ (P) enzymes were provided by DuPont (Buenos Aires, Argentina) and Nutring (Buenos Aires, Argentina), respectively. Flavourzyme (F), pepsin, and pancreatin enzyme was obtained from Sigma Chemical Co. (St. Louis, MO, USA). The other reagents were obtained from Sigma-Aldrich (Madrid, Spain). In vitro experiments were carried out with LPS from *Escherichia coli* 055:B5. Rat ELISA kits (IL-10 and TNF) were obtained from BD Pharmingen (San Agustín, Spain), except for IFN-γ (R&D Systems, Minneapolis, MN, USA). Mouse ELISA kits (IL-10, TNF) were obtained from eBioscience (San Diego, CA, USA).

### 4.2. Raw Materials

One kilogram of different specimens of *Ulva* spp. was handpicked in Punta Maqueda (Comodoro Rivadavia, Argentina). The seaweed was processed according to Cian et al. [31].

### 4.3. Preparation of Hydrolysates

*Ulva* spp. hydrolysates and their peptide fractions were prepared as shown in Supplementary Figure S2. The chlorophyll *a* and *b* from *Ulva* spp. was extracted [32] and green marine alga *Ulva* spp. was dispersed at 25 g kg^−1^ in acetone for 30 min and filtered through a 50-mesh sieve (0.297 mm). The residue was re-extracted four times. To remove solvent remains, the residue was dried using DFZ-6020 (Zenithlab, Pomona, CA, USA) vacuum oven at 40 °C for 3 h. The dry residue obtained from chlorophyll extraction was used as substrate for enzymatic hydrolysis.

Hydrolysates were obtained using an 800 mL batch thermostatic reactor. The reaction pH was continuously measured using pHmeter IQ Scientific Instruments (Carlsbad, CA, USA), and adjusted by adding base (NaOH, 2 mol L^−1^) or acid (HCl, 2 mol L^−1^) with a burette. Substrate concentration was 1 g 100 g^−1^ dispersion. Working conditions were: temperature 55 °C, pH 9.5, enzyme/substrate (E/S) ratio 5 g 100 g^−1^; temperature 50 °C, pH 7.0, enzyme/substrate (E/S) ratio 5 g 100 g^−1^; and temperature 55 °C, pH 7.0, enzyme/substrate (E/S) ratio 5 g 100 g^−1^, for A, P, and F respectively. Once the hydrolysis was finished, the enzymes were inactivated by thermal treatment following the manufacturer’s guidelines and the hydrolysates were centrifuged at 2000× *g* for 15 min at 4 °C. The supernatant obtained was lyophilized. *Ulva* spp. hydrolysates were prepared using the following systems:

- Hydrolysate PF: hydrolysis with P enzyme 2 h + hydrolysis with F enzyme during 2 h; total reaction time, 4 h.
- Hydrolysate AF: hydrolysis with A enzyme 2 h + hydrolysis with F enzyme during 2 h; total reaction time, 4 h.

Free amino groups were measured using o-phthaldialdehyde, according to Nielsen, Petersen, and Dambmann [33], and the degree of hydrolysis (DH) was calculated as:

DH = [(h − h_0_)/h_tot_] × 100 (1)

where h_tot_ is the total number of peptide bonds in the protein substrate (8.04 mEq g^−1^ protein); h is the number of peptide bonds cleaved during hydrolysis, and h_0_ is the content of free amino groups of substrate.

### 4.4. In Vitro Gastrointestinal Digestion of Hydrolysates

The hydrolysates were dispersed at 4 g L^−1^ of protein. Aliquots (100 mL) of hydrolysates dispersions were adjusted to pH 2.0 with 4 mol L^−1^ of HCl and after addition of 0.8 mL pepsin digestion mixture (16 g 100 mL^−1^ pepsin solution in 0.1 mol L^−1^ HCl), were incubated at 37 °C for 2 h in a shaking water bath. After pepsin hydrolysis, the pH was gradually increased to 7.0 with 1 mol L^−1^ of NaHCO_3_, during 10 min. Then, 6.25 mL of a pancreatin solution (0.4 g 100 mL^−1^ in 0.1 mol L^−1^ NaHCO_3_) was added and the dispersion incubated for 2 h at 37 °C. Digested products were immediately placed in boiling water for 4 min to inactivate the enzymes. Then, the products were centrifuged at 3500 g for 30 min at room temperature and the supernatant lyophilized. Digested products from PF and AF were named PFD and AFD, respectively. PFD and AFD were fractionated by ultrafiltration as detailed below.

### 4.5. Fractionation of Hydrolysates and Digested Hydrolysates

The hydrolysates and digested hydrolysates were ultrafiltered using a 1 kDa cut-off Molecular/Por Cellulose-Ester membrane and Molecular/Por Stirred Cell S-43-70 system. The volume reduction factor was 3. The fractions with molecular weight <1 kDa were lyophilized and named PFU, AFU, PFDU, and AFDU.

### 4.6. Fast Protein Liquid Chromatography (FPLC) of Hydrolysates and Ultrafiltered Fractions (<1 kDa)

Gel filtration chromatography was carried out in an AKTA Prime system equipped with a Superdex peptide column (GE Life Sciences, Piscataway, NJ, USA). Injection volume was 100 mL (2.8 mg protein mL^−1^) and elution was carried out using 50 mmol L^−1^ potassium phosphate buffer pH 7.0 plus 150 mmol L^−1^ NaCl at 1 mL min^−1^. Elution was monitored at 280 nm and molecular mass was estimated using molecular weight (MW) standards from Pharmacia Fine Chemicals (Piscataway, NJ, USA): blue dextran (2,000,000 Da), cytochrome C (12,500 Da), aprotinin (6512 Da), bacitracin (1450 Da), cytidine (246 Da) and glycine (75 Da).

### 4.7. Animals

Female Wistar rats (175–225 g) were obtained from Janvier Labs (Le Genest-Saint Isle), while mice (male C57BL/6J wild type, B6.B10ScN-Tlr4lps-del/JthJ (Tlr4 KO) and B6.129-Tlr2 < tm1Kir >/*J* (Tlr2 KO)) were obtained from Jackson Laboratory (CA, USA). Animals were maintained at the unit of animal research (Biomedical Research Center, University of Granada, Granada, Spain) in air-conditioned animal quarters with a 12 h light-dark cycle in specific pathogen-free (SPF) conditions, with free access to autoclaved tap water and food (Harlan-Teklad 2014, Harlan Ibérica, Barcelona, Spain). This study was in accordance with Directive 2010/63/EU and approved by the ethical committee (ref. ES 01/03/2017/029, Granada, Spain).

### 4.8. Spleen Mononuclear Cells Ex Vivo Culture

Spleens were extracted using sterile technique and dissected mechanically as described [34] and cultured in RPMI-1640 medium containing fetal bovine serum (10%). Cell viability was quantified with the Trypan blue exclusion assay. T cells and macrophages were purified from rat splenocytes by magnetic column negative selection (anti-CD11b, anti-CD161, anti-CD45RA and anti-CD3, anti-CD161, anti-CD45RA, respectively) with materials provided by Miltenyi Biotec (Cologne, Germany). Purity of the enriched population was verified by flow cytometry (FACSCalibur; BD Biosciences, San Jose, CA, USA).

### 4.9. Protein Determination

Protein content of hydrolysates and their fractions was determined by bincinchoninic acid method using bovine serum albumin as standard [35].

### 4.10. Cytokine Determination

Cells suspension (10^6^ cells mL^−1^) were cultured in the presence or absence of product (1, 0.1 and 0.01 g L^−1^ of protein) and stimulated with ConA (5 µg mL^−1^) or LPS (1 µg mL^−1^), depending on the cell type. Cell culture medium was collected after 24 or 48 h, cleared by centrifugation (2000× *g*, 10 min, and 4 °C) and frozen at −80 °C until cytokine determination by commercial ELISA kits. In all the experiments, samples were run in triplicate and results are expressed as cytokine concentration (pg mL^−1^).

### 4.11. Cell Toxicity Assay

Cell toxicity was measured in cell culture supernatant using Pierce LDH cytotoxicity assay kit (Thermo Fisher Scientific, Waltham, MA, USA).

### 4.12. NFκB and Mitogen-Activated Protein Kinase (MAPK) Inhibitors Assay

To explore downstream signaling pathways, rat splenocytes were exposed to Bay 11-7082 (10 μM), a selective inhibitor of IκB-α phosphorylation that blocks the NFκB signaling pathway, or the MAPK inhibitors SB203580 (p38 MAPK inhibitor, 10 μM), PD98059 (ERK1/2, 10 μM) (EMD Millipore, Billerica, MA, USA), or SP600125 (JNK, 10 μM). All the inhibitors were dissolved in DMSO and were added to the culture medium 1 h before the challenge with the different products (without further stimulation).

### 4.13. Data and Statistical Analysis

All results are expressed as mean ± SD. The data were analyzed by one-way analysis of variance, using the software STATGRAPHICS Centurion XV 15.2.06 (Statpoint Technologies, Inc., Warrenton, VA, USA). The statistical differences between samples were determined using the LSD (least significant difference) test on preselected pairs. The significance was established at *P* < 0.05.

## Supplementary Materials

The following are available online at http://www.mdpi.com/1660-3397/16/7/235/s1, Figure S1: FPLC gel filtration profiles of *Ulva* spp. hydrolysates, their ultra-filtered fractions, and gastrointestinal digested hydrolysates followed by ultrafiltration. Figure S2: Processing diagram to obtain *Ulva* spp. hydrolysates and their peptide fractions. Figure S3: Effect of trichloroacetic acid supernatant of *Ulva* spp. hydrolysate products on the production of IL-10 and TNF by rat splenocytes.

## Abbreviations

AF	hydrolysate obtained with Alcalase + Flavourzyme
AFU	AF fractions with molecular weight <1 kDa
AFDU	fractions with molecular weight <1 kDa from in vitro gastrointestinal digestion of AF
PF	hydrolysate obtained with Purazyme + Flavourzyme
PFDU	fractions with molecular weight <1 kDa from in vitro gastrointestinal digestion of PF
PFU	PF fractions with molecular weight <1 kDa
TLR	Toll-like receptors
