# Supplementary figures and images for: Green Alga Ulva spp. Hydrolysates and Their Peptide Fractions Regulate Cytokine Production in Splenic Macrophages and Lymphocytes Involving the TLR4-NFκB/MAPK Pathways
Source: Mar Drugs. 2018 Jul 11;16(7):235. doi: 10.3390/md16070235 (PMC6071126; doi:10.3390/md16070235)

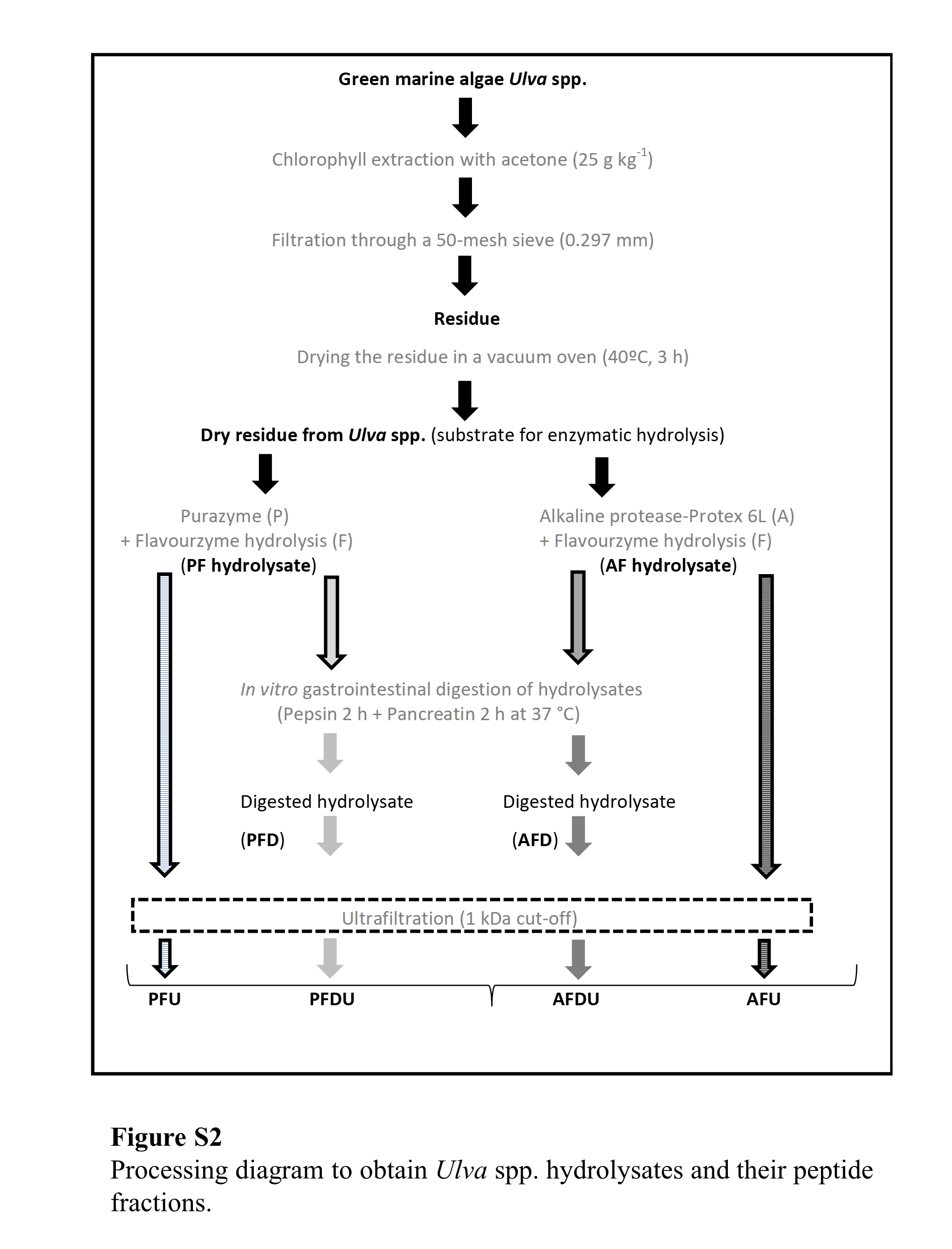

Supplement: Supplementary file 1 [file marinedrugs-16-00235-s001.zip › Figure S2.tif]

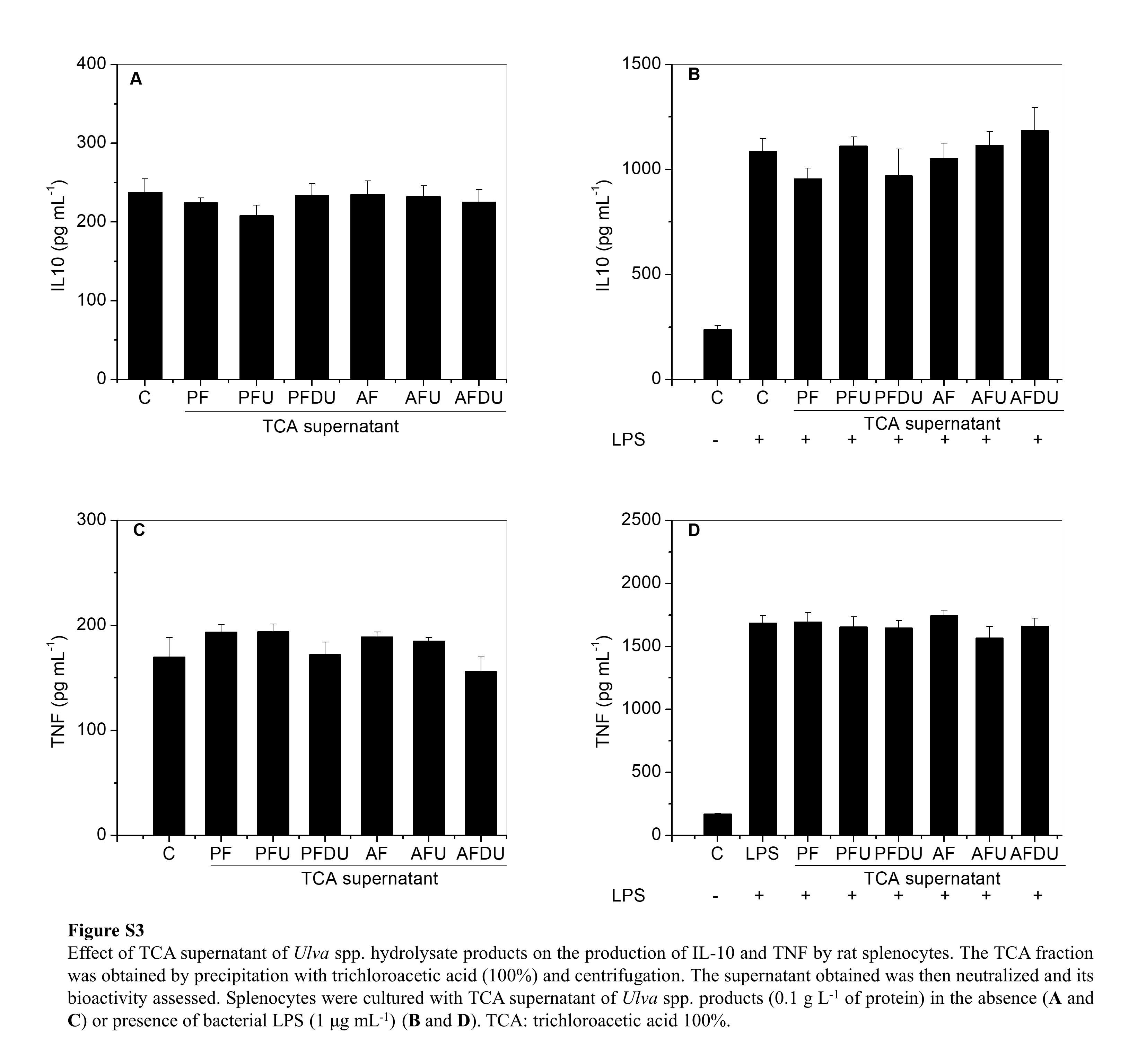

Supplement: Supplementary file 1 [file marinedrugs-16-00235-s001.zip › Figure S3.tif]

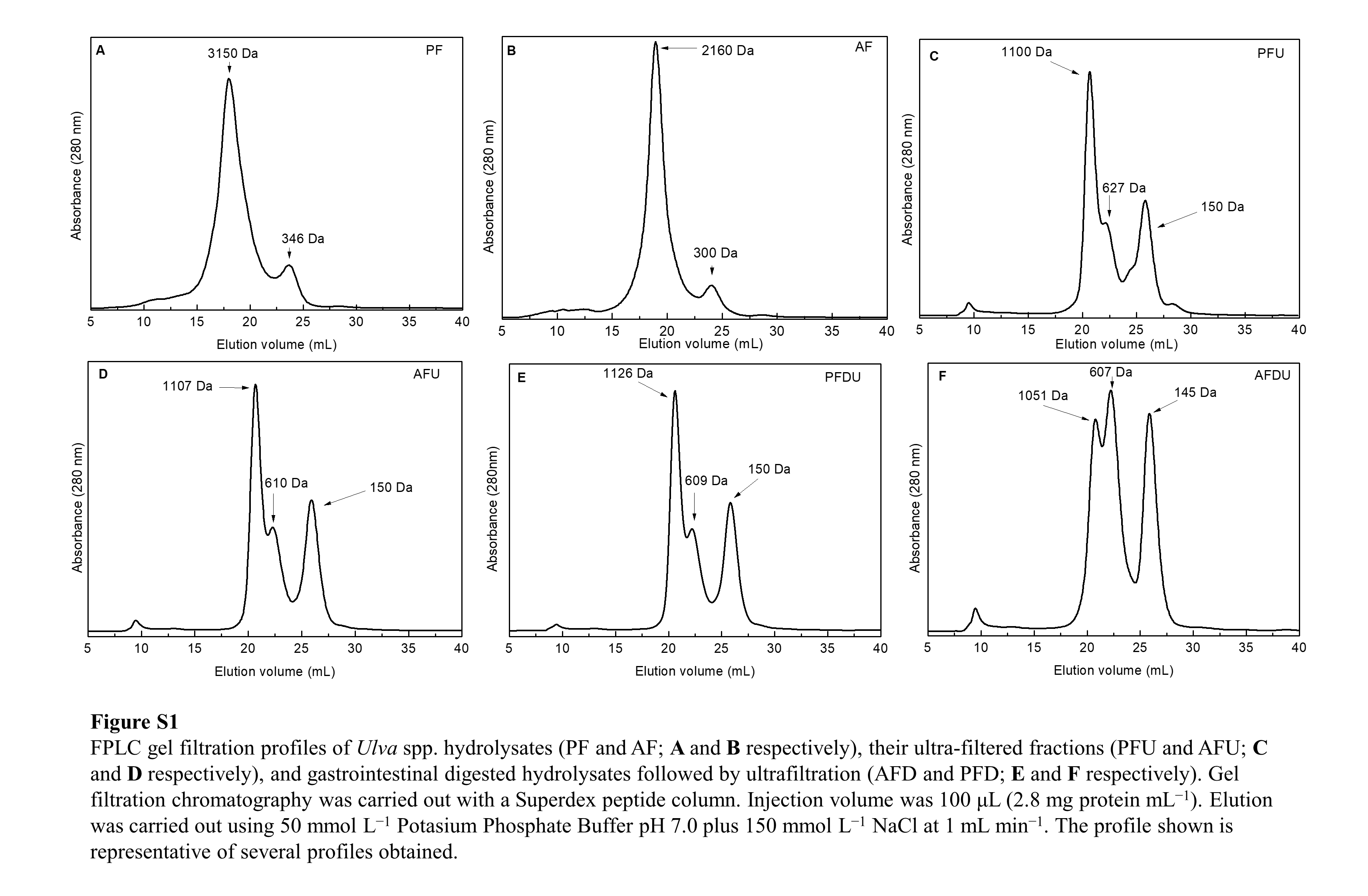

Supplement: Supplementary file 1 [file marinedrugs-16-00235-s001.zip › Figure S1.tif]
